# Gait Apraxia with Exaggerated Upper Limb Movements as Presentation of AARS2 Related Leukoencephalopathy

**DOI:** 10.5334/tohm.705

**Published:** 2022-08-02

**Authors:** Arka Prava Chakraborty, Adreesh Mukherjee, Aishee Bhattacharyya, Dwaipayan Bhattacharyya, Biman Kanti Ray, Atanu Biswas

**Affiliations:** 1 Department of Neurology, Institute of Post Graduate Medical Education & Research and Bangur Institute of Neuroscience, Kolkata, India

**Keywords:** AARS2 mutation, gait apraxia, familial, leukodystrophy, MRI

## Abstract

A 55-year-old male presented with apraxia of gait with exaggerated upper limb movement with relative preservation of cognition and mild spasticity of limbs. His investigations reveal posterior-predominant leukodystrophy in brain magnetic resonance imaging (MRI) and compound heterozygous mutations in mitochondrial alanyl-transfer RNA synthetase 2 (*AARS2*) by next generation sequencing. His asymptomatic brother also has MRI changes with subtle mild pyramidal signs. *AARS2* mutation is a rare cause of mitochondrial encephalopathy which may give rise to leukodystrophy with premature ovarian failure, infantile cardiomyopathy, lung hypoplasia and myopathy. Gait apraxia as primary presenting feature of this rare variant of mitochondrial encephalomyopathy is hitherto un-reported.

## Introduction

Biallelic variants in mitochondrial alanyl-transfer RNA synthetase 2 (*AARS2*) cause a form of mitochondrial encephalomyopathy which may give rise to leukodystrophy with premature ovarian failure, infantile cardiomyopathy, lung hypoplasia and myopathy [1]. They may also present with cognitive, pyramidal, extrapyramidal and cerebellar dysfunction. Only few cases have been described worldwide so far. We present a subject with AARS2 related leukodystrophy, one of them presenting with gait apraxia whose brother also has MRI changes with subtle clinical features.

## Case Report

A 55-years-old gentleman presented with chronic progressive course of walking difficulty for 8 years and dysarthria for 3 years. He also complained of heaviness and stiffness of both lower limbs while walking. He was taking multiple stuttering in-place steps before starting, and was walking in broad base with hesitant steps with difficulties in turning. He used bizarre dance-like bilateral upper limb movements to compensate and steer forward (Video 1). The problem increased when he was given a mental task like counting backwards from 100. A graduate, he was born of non-consanguineous marriage of Indian parentage with normal birth and developmental history.

**Video 1:** **Gait examination.** Showing difficulty in initiation of gait with multiple stuttering of steps associated dance-like bilateral upper limb movements. The gait is broad base with hesitant steps with difficulties in turning.

**Figure 1:**
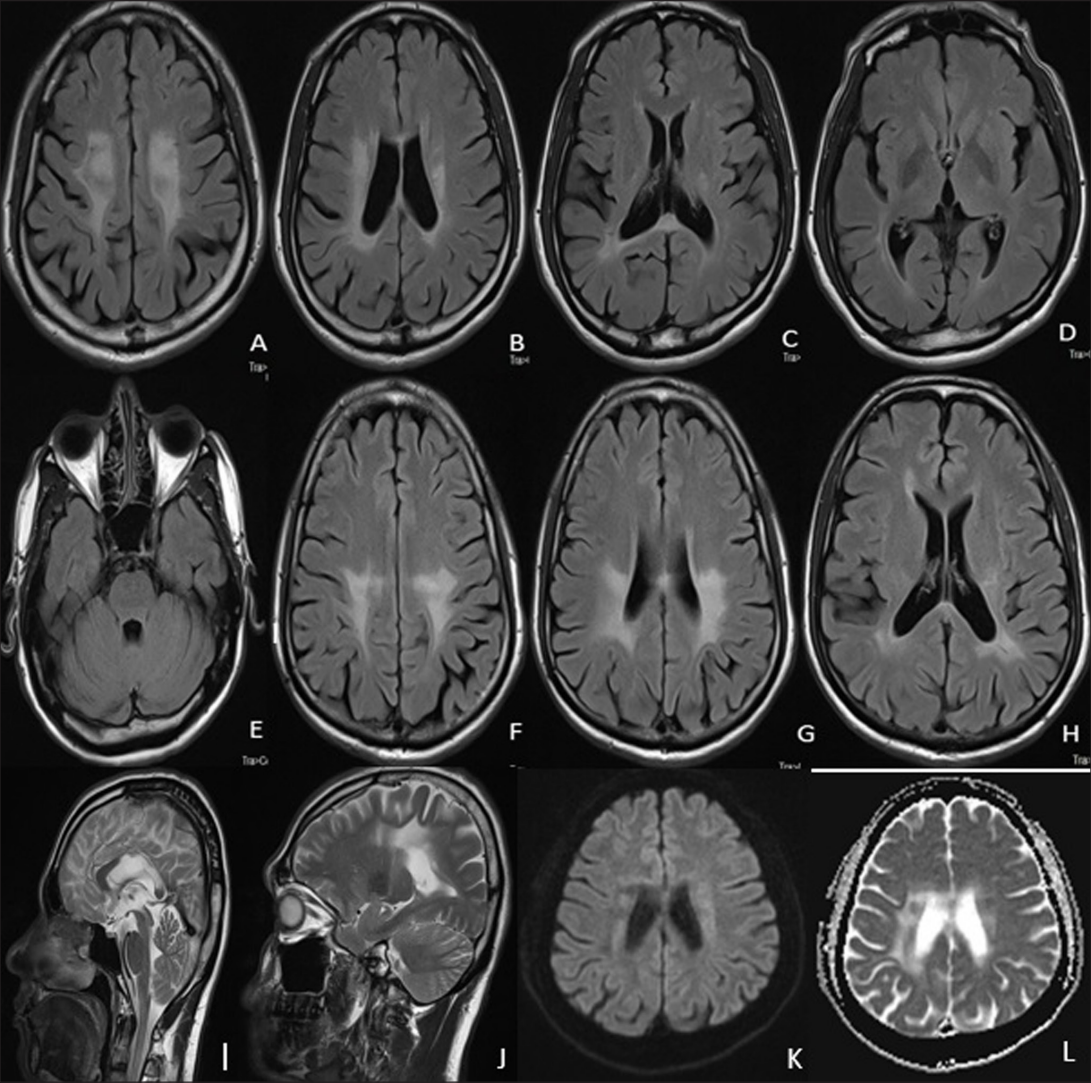
**1A–1E:** T2 FLAIR MR image showing diffuse bilateral white matter hyperintensities involving bilateral centrum semiovale, periventricular regions, splenium and forceps major, sparing frontal white and deep grey matter, brainstem and cerebellum. **1F–1H:** T2 FLAIR images in patient’s brother with similar changes, albeit to a lesser extent. **1I–1J:** T2 sagittal MR image shows corpus callosal body and splenial involvement along with parieto-occipital subcortical involvement. **1K–1L:** Diffusion weighted imaging shows faint DW restriction in periventricular area with shine through on ADC (apparent diffusion coefficient) map.

He scored 82 in Addenbrooke’s Cognitive Examination and 15 in Frontal Assessment Battery. He showed mild attentional deficit and poor delayed recall in word list memory task, that improved with cues with preserved other cognitive domains. His examination revealed slurred speech, mild spasticity of four limbs, normal limb power with brisk deep tendon reflexes (DTRs). He could perform complicated coordinated motor activities by lower limbs while lying down like drawing numbers in the air or cycling in midair (Video 2). However, his gait problem did not improve with visual cues. His MRI (magnetic resonance imaging) of brain showed bilateral posterior predominant periventricular white matter hyperintensities in T2 and FLAIR sequences suggestive of leukodystrophy (Figure 1A-E and 1I-L). Investigations including complete blood count, blood biochemical parameters, serum lactate, creatinine phosphokinase, electrocardiogram and 2D echocardiogram were normal. Tests for arylsulphatase A, urinary metachromatic granules and very long chain fatty acids came negative. There was no family history, but on examination, his brother was found to have dysarthria with mild spasticity and brisk DTRs in lower limbs. His brain MRI also showed posterior predominant white matter hyperintensities (Figure 1 F–H). Clinical exome of the index case by next generation sequencing revealed likely compound heterozygous mutations of the AARS2 gene namely NM_020745.4 (*AARS2*):c.1874G > A (p.Arg625His) and NM_020745.4 (*AARS2*):c.179C > A (p.Pro60His) which were classified as being “likely pathogenic”. Parental study could not be performed in this patient and thus there was no data regarding trans or cis position of the mutated alleles in the compound heterozygous state. In-silico prediction found both the variants to be damaging. No other variants in any gene causing leukodystrophy were found. He was put on coenzyme complex therapy and other supportive treatment, and is now under follow-up. His brother denied permission for genetic testing.

**Video 2:** **Leg movements in other tasks.** The subject is performing complicated coordinated motor activities with his lower limbs while lying down like cycling in midair or drawing numbers in the air.

## Discussion

Superfluous compensatory upper limb movements have been described for gait apraxia [2]. In the index case, such movements were thought to be primarily choreiform, but on close observation the gait difficulty was found to be the primary abnormality and upper limb movements were compensatory. His gait was due to ignition failure and his feet were “glued” to the floor, difficult for him to make steps and his upper limbs were moving in a dance-like fashion to compensate restricted movement of his feet. His gait difficulty was increasing when he was given a mental task. This led us to clinically label it as gait apraxia. Freezing of gait and apraxia of gait have been earlier described as clinically distinct entities with some overlap. Unlike gait apraxia, freezing of gait is intermittent and responds well with visual cues. Furthermore, there is presence of bizarre compensatory limb movements in gait apraxia, which is seldom encountered in freezing of gait [3]. Present case showed no improvement with visual cues, there was upper limb compensatory movements and was not intermittent. This weighed in favour of gait apraxia rather than pure freezing of gait. In absence of gross spasticity, weakness or cerebellar signs in the lower limbs with preserved ability to perform complicated motor acts [4] such as drawing numbers with legs or cycling in midair, our diagnosis was further substantiated. Localization of gait apraxia is usually in medial frontal cortex, paracentral lobule or supplementary motor area (SMA) [5]. Gait apraxia has also been reported in subjacent white matters of SMA region [6] and in periventricular white matter lesions in patients with multiple sclerosis [7]. Disruption of periventricular white matter tracts connecting brainstem gait centres to SMA or paracentral lobule, can be a plausible explanation of gait apraxia in our patient, implicating involvement of complex neural networks [6] rather than particular areas in control of gait.

AARS2 mutations lead to patchy leukodystrophy mainly in frontal and parietal periventricular white matter with combination of cognitive impairment with frontal lobar dysfunction, spasticity and ataxia [8] and female patients having premature ovarian failure. Only about 46 cases have been described worldwide [9]. MRI abnormalities may be along tracts and involving different parts or whole of the corpus callosum. In contrast, though both of our patients had involvement of splenium, they had sparing of frontal subcortical white matters. Gait difficulties in previously described patients were mainly due to spasticity and ataxia. But gait apraxia as the presenting and dominant gait problem, makes our case special. There was no cardiac problem or evidence of myopathy. Comparing previous cases [10] (mean age ~ 25 years), our patient had the highest reported age and showing familial occurrence of this disease. Tempo of the disease was also slow in our case as compared to previous reports of rapid progression after diagnosis. NM_020745.4 (*AARS2*):c.179C > A (p.Pro60His) AARS2 variant has been previously reported in a patient with leukodystrophy with weakness and numbness of right lower limb with aphasia, cognitive decline and dysarthria [10]. Inability to do familial testing of AARS2 gene remains as a limitation of our study.

To conclude, gait apraxia as primary presenting feature of this rare variant of mitochondrial encephalomyopathy, makes our case noteworthy.

## References

[1] Axelsen TM, Vammen TL, Bak M, Pourhadi N, Stenør CM, Grønborg S. Case report: ‘AARS2 leukodystrophy’. Mol Genet Metab Rep. 2021 Jul 13; 28: 100782. PMID: 34285876; PMCID: PMC8280508. DOI: 10.1016/j.ymgmr.2021.100782 34285876 PMC8280508

[2] Della Sala S, Spinnler H, Venneri A. Walking difficulties in patients with Alzheimer’s disease might originate from gait apraxia. J Neurol Neurosurg Psychiatry. 2004 Feb; 75(2): 196–201. PMID: 14742586; PMCID: PMC1738895. 14742586 PMC1738895

[3] Dale ML, Curtze C, Nutt JG. Apraxia of gait- or apraxia of postural transitions? Parkinsonism Relat Disord. 2018 May; 50: 19–22. Epub 2018 Feb 19. PMID: 29477458. DOI: 10.1016/j.parkreldis.2018.02.024 29477458

[4] Rajput A, Rajput A. Old age and Parkinson’s disease. In: Handbook of Clinical Neurology. 2007; 427–444. DOI: 10.1016/S0072-9752(07)84053-X 18808962

[5] Della Sala S, Francescani A, Spinnler H. Gait apraxia after bilateral supplementary motor area lesion. J Neurol Neurosurg Psychiatry. 2002 Jan; 72(1): 77–85. PMID: 11784830; PMCID: PMC1737704. DOI: 10.1136/jnnp.72.1.77 11784830 PMC1737704

[6] Nadeau SE. Gait apraxia: further clues to localization. Eur Neurol. 2007; 58(3): 142–5. Epub 2007 Jun 29. PMID: 17622719. DOI: 10.1159/000104714 17622719

[7] Abou Zeid NE, Weinshenker BG, Keegan BM. Gait apraxia in multiple sclerosis. Can J Neurol Sci. 2009 Sep; 36(5): 562–5. PMID: 19831123. DOI: 10.1017/S0317167100008040 19831123

[8] Dallabona C, Diodato D, Kevelam SH, et al. Novel (ovario) leukodystrophy related to *AARS2* mutations. Neurology. 2014; 82(23): 2063–2071. DOI: 10.1212/WNL.0000000000000497 24808023 PMC4118500

[9] Zhang X, Li J, Zhang Y, Gao M, Peng T, Tian T. AARS2-Related Leukodystrophy: a Case Report and Literature Review. Cerebellum. 2022 Jan 27. DOI: 10.1007/s12311-022-01369-5 35084689

[10] Tang Y, Qin Q, Xing Y, Guo D, Di L, Jia J. AARS2 leukoencephalopathy: A new variant of mitochondrial encephalomyopathy. Mol Genet Genomic Med. 2019 Apr; 7(4): e00582. Epub 2019 Jan 31. PMID: 30706699; PMCID: PMC6465728. DOI: 10.1002/mgg3.582 30706699 PMC6465728

